# miR-18a and miR-106a Signatures in Plasma Small EVs Are Promising Biomarkers for Early Detection of Pancreatic Ductal Adenocarcinoma

**DOI:** 10.3390/ijms24087215

**Published:** 2023-04-13

**Authors:** Xiaohui Xu, Kritisha Bhandari, Chao Xu, Katherine Morris, Wei-Qun Ding

**Affiliations:** 1 Department of Pathology, University of Oklahoma Health Sciences Center, Oklahoma City, OK 73104, USA; xhxu_tc1988@suda.edu.cn (X.X.);; 2 Department of Biostatistics & Epidemiology, University of Oklahoma Health Sciences Center, Oklahoma City, OK 73104, USA; 3 Department of Surgery, University of Oklahoma Health Sciences Center, Oklahoma City, OK 73126, USA

**Keywords:** extracellular vesicles, pancreatic cancer, microRNAs, biomarkers, early detection

## Abstract

Pancreatic cancer is the third leading cause of cancer-related death in the United States. Pancreatic ductal adenocarcinoma (PDAC) is the major form of pancreatic cancer with the worst outcomes. Early detection is key to improving the overall survival rate of PDAC patients. Recent studies have demonstrated that microRNA (miRNA) signatures in plasma small extracellular vesicles (EVs) are potential biomarkers for the early detection of PDAC. However, published results are inconsistent due to the heterogeneity of plasma small EVs and the methods used for small EV isolation. We have recently refined the process of plasma small EV isolation using double filtration and ultracentrifugation. In the present study, we applied this protocol and analyzed plasma small EV miRNA signatures by small RNA sequencing and quantitative RT-PCR in a pilot cohort, consisting of patients with early-stage PDAC, and age- and gender-matched healthy subjects (n = 20). We found, via small RNA sequencing, that there are several miRNAs enriched in plasma small EVs of PDAC patients, and the levels of miR-18a and miR-106a were confirmed by quantitative RT-PCR to be significantly elevated in patients with early-stage PDAC compared with age- and gender-matched healthy subjects. Furthermore, using an immunoaffinity-based plasma small EV isolation approach, we confirmed that the levels of miR-18a and miR-106a in plasma small EVs were significantly higher in PDAC patients versus the healthy subjects. We thus conclude that the levels of miR-18a and miR-106a in plasma small EVs are promising biomarkers for the early detection of PDAC.

## 1. Introduction

Approximately 62,000 new cases of, and more than 49,000 deaths from, pancreatic cancer were expected in 2022. The 5-year overall survival rate for pancreatic cancer is lower than other solid tumor types, with a 37% survival rate for localized tumors (stage I–IIA), 12% for cases with regional lymph node involvement (stages IIB and III), and 3% for distally metastasized patients (stage IV) [1]. The major form and the most lethal type of pancreatic tumors is pancreatic ductal adenocarcinoma (PDAC). Late-stage diagnoses of PDAC correlate with a high mortality rate, as clinical symptoms have manifested and treatment options are limited [2,3]. Several factors contribute to the difficulty of early detection of PDAC: (1) there are no signs or symptoms in the early stages; (2) the signs of PDAC, when they do occur, are often not specific to the disease; (3) the pancreas is obscured by other organs, which compromises imaging tests [2,3]. Early detection through circulating biomarkers is key to improving PDAC outcomes. However, there are no sensitive and specific circulating biomarkers for the early detection or timely monitoring of PDAC. The current plasma biomarkers for PDAC, such as carbohydrate antigen 19-9 (CA19-9) and carcinoembryonic antigen (CEA), are neither sensitive nor specific for the screening of this malignancy [3].

MicroRNA (miRNA) expression is significantly deregulated in PDAC cells and tissues [4,5]. Consequently, circulating miRNA signatures have been explored to define new biomarkers for PDAC [6]. Plasma exosome miRNA signatures suggestive of PDAC have been recently reported [7,8,9,10,11], and we have observed that some miRNA species, such as miR-196a and miR-21, are highly enriched in cancer exosomes [12,13]. These enriched exosome miRNAs are present at significantly higher levels in plasma exosomes obtained from early-stage PDAC and breast cancer patients [12,13]. These findings support the idea that plasma exosome miRNA signatures are promising biomarkers for the early detection or monitoring of PDAC.

However, the sensitivity and specificity of plasma cancer exosome miRNA detection is challenged by at least two inherent variables. First, miRNA contents differ among various types of EVs [14], and the commonly used exosome isolation methods, such as ultracentrifugation or commercial reagents, often result in a mixed population of EVs [15]. Second, the plasma contains EV populations released from various cell types, such as blood cells and endothelial cells [16], and cancer EVs are included in the mixture. These two inherent factors inevitably compromise the usefulness of plasma small EV miRNA detection in cancer patients, which would explain why there are no clinically applicable plasma EV miRNA signatures for cancer detection, even though many studies have shown their promise [17]. New approaches are needed to overcome these challenges in the development of plasma small EV miRNA signatures as biomarkers for PDAC. To counter the first inherent variable, we have developed a new protocol using double filtration and ultracentrifugation to collect plasma small EVs at sizes around 100 nm in diameter, ensuring a more uniform population of exosome vesicles [18]. In the present study, we demonstrate by small RNA sequencing followed by qRT-PCR that the levels of miR-18a and miR-106a are significantly elevated in size-restricted plasma small EVs from patients with early-stage PDAC (stage I–IIA) compared with gender- and age-matched healthy controls. To overcome the second inherent variable, we have modified an established immunoaffinity isolation strategy [8,19] that enables us to selectively enrich PDAC exosomes from patient plasma for miRNA analysis. By applying a magnetic-bead-conjugated antibody cocktail, we immunoprecipitated plasma small EVs and found that the levels of miR-18a and miR-106a are significantly elevated in plasma small EVs from patients with early-stage PDAC compared to healthy controls, in line with our findings using size-restricted plasma small EVs.

## 2. Results

### 2.1. Several miRNAs Are Significantly Enriched in the Small EVs Derived from PDAC Patient Plasma

To take an unbiased approach, we performed small RNA sequencing using the Illumina platform SE50 on plasma small EVs collected from patients with early-stage PDAC (stage I–IIA, Table 1) and age- and gender-matched healthy subjects. Among 243 miRNAs detected by small RNA sequencing, the levels of 4 miRNAs, including miR-106a, miR-18a, miR-502, and miR-660, were significantly elevated in PDAC plasma small EVs compared to those in the plasma of matched healthy subjects (Table 2). While higher reads of miR-21 were also observed in PDAC plasma small EVs, there was no statistical difference between the two groups, as judged by the BH adjusted *p*-value.

### 2.2. qRT-PCR Confirms the Elevated Levels of miR-18a and miR-106a in Plasma Small EVs Derived from PDAC Patients

**Figure 1:**
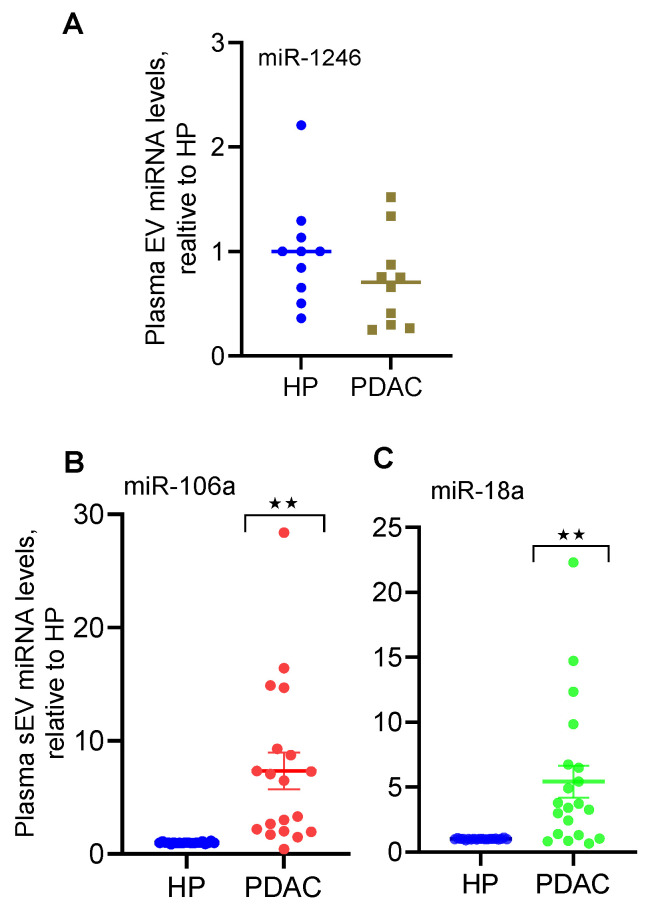
Elevated expression of selected miRNAs in small EVs isolated from PDAC patient plasma. Small EVs were isolated from plasma (250 μL each) of patients with early-stage PDAC and age- and gender-matched healthy subjects (HP). miRNA expression was analyzed by qRT-PCR. Expression of miR-1246 ((**A**), n = 10), miR-106a ((**B**), n = 19), and miR-18a ((**C**), n = 20) is shown. ** *p* < 0.01, paired Student’s *t*-test.

qRT-PCR analysis ([20,21], Table 3) verified that the levels of miR-18a and miR-106a were significantly elevated in small EVs derived from PDAC patient plasma compared with the plasma from age- and gender-matched healthy controls (Figure 1). The levels of miR-502 in plasma small EVs were barely detectable by qRT-PCR both in patient plasma and healthy plasma, whereas miR-1246 expression remained the same in both groups of small EVs. The levels of miR-660 were not analyzed by qRT-PCR considering that its fold change was the smallest among these miRNAs.

### 2.3. The Levels of miR-18a and miR-106a Are Significantly Elevated in Immunoprecipitants of Plasma Small EVs from PDAC Versus Those from Colon Cancer and Healthy Controls

Recent studies have identified five membrane proteins expressed on the surface of PDAC plasma exosomes, and applying antibodies against these five proteins for immunoaffinity isolation enriched PDAC exosomes from patient plasma [8,19]. Antibodies against these five proteins (human CD326 (EpCAM, BioLegend), CD104, c-Met, CD44v6 (Thermo Fisher Scientific), and TSPAN8 (Miltenyi Biotec)) were conjugated to magnetic beads (Dynabeads Protein G, Life Technologies). Using the magnetic beads conjugated to antibodies, plasma EVs were isolated following the procedures we described previously [12]. Total RNA was isolated from the immunoprecipitated plasma small EVs. qRT-PCR analysis demonstrated that the levels of miR-18a and miR-106a, but not miR-196a, were significantly elevated in the immunoprecipitants of plasma small EVs from PDAC patients compared with those from healthy controls and colon cancer patients (Table 4 and Table 5), further indicating that miR-18a and miR-106a signatures in plasma small EVs are promising biomarkers for the early detection of PDAC.

## 3. Discussion

miRNA signatures in patient plasma have been investigated as potential biomarkers for PDAC [6], and plasma exosome miRNA signatures suggestive of PDAC have been described [7,8,9,10,11]. However, there has been no consensus as to which set of plasma miRNA signatures are better indicators of PDAC. An applicable clinical test for PDAC using plasma miRNA signatures is currently unavailable, suggesting that new approaches are necessary to explore the potential of plasma miRNA signatures as non-invasive biomarkers for PDAC. Using our newly developed protocol for small EV isolation [18], the present study demonstrates that miRNA signatures in plasma small EVs are promising biomarkers for the early detection of PDAC. Specifically, the levels of miR-18a and miR-106a in plasma small EVs were significantly elevated in patients with early-stage PDAC compared with matched healthy controls. These observations were further confirmed by immunoaffinity isolation of plasma small EVs, supporting the development of miR-18a and miR-106a signatures in plasma small EVs as indicators for early-stage PDAC.

Our small RNA sequencing effort led to the identification of four miRNAs, including miR-18a, miR-106a, miR-502, and miR-660, whose levels were significantly elevated in plasma small EVs derived from patients with early-stage PDAC (Table 1) compared with matched healthy controls. Immunoaffinity isolation and qRT-PCR analysis confirmed that the levels of miR-18a and miR-106a were significantly elevated in plasma small EVs from PDAC patients; however, miR-502 was barely detectable in plasma small EVs both from PDAC patients and from matched healthy controls. The discrepancy of small RNA sequencing versus qRT-PCR results in miR-502 expression could be due to the technical difference between small RNA sequencing and qRT-PCR, and these results suggest that the levels of miR-18a and miR-106a in plasma small EVs are more reliable indicators than the level of miR-502 for early-stage PDAC. Plasma miR-106a levels have been previously reported to be indicative of gastric cancer [22], colon cancer [23], and prostate cancer [24]. Likewise, plasma miR-18a levels have been demonstrated to be elevated in patients with pancreatic cancer [25] and other types of cancer [26]. To our knowledge, however, miR-18a and miR-106a levels in plasma small EVs have not been previously explored as potential circulating biomarkers for human cancer, and there have been no reports on the potential of plasma miR-18a and miR-106a signatures for the early detection of PDAC. The present study thus provides strong evidence supporting the development of miR-18a and miR-106a signatures in plasma small EVs as non-invasive biomarkers for the early detection of PDAC.

We have previously reported that the level of miR-196a in plasma small EVs is elevated in patients with early-stage PDAC [13,18] in a small patient cohort. The dysregulation of miR-196a expression in pancreatic cancer tissues has been described [27]. However, the analysis of the immunoaffinity-isolated plasma small EVs failed to detect significant changes in miR-196a levels, further suggesting that relative to the levels of miR-196a, miR-18a and miR-106a levels in plasma small EVs are more reliable indicators of early-stage PDAC. These observations support the conclusion that the levels of miR-18a and miR-106a in plasma small EVs are promising indicators of early-stage PDAC. This conclusion needs to be verified through future large-cohort studies.

## 4. Materials and Methods

Patient plasma. Patient plasma samples were obtained from the NCI-sponsored Cooperative Human Tissue Network (CHTN), which collects patient plasma samples following standard protocols. Plasma from colon cancer patients was collected at the Stephenson Cancer Center, and healthy plasma was provided by the Oklahoma Blood Institute, following an approved IRB protocol (IRB#: 12781). Pathological diagnosis, age, gender, and race of the patients were provided with the plasma specimens (Table 1 and Table 5).

Small EV isolation. Plasma small EVs were isolated using a protocol we recently published with minor modifications [18]. Briefly, plasma (200 µL) was centrifuged at 3000× *g* for 5 min at room temperature to remove any potential debris. The resulting supernatant was treated with thrombin (5 units/mL, Millipore Sigma) for 5 min at room temperature, and the sample was centrifuged at 10,000× *g*, at room temperature, for another 5 min. The collected supernatant was subjected to a double filtration process as previously described [18]. After the second filtration, small EVs were recovered either by ultracentrifugation (100,000× *g*, 90 min) or using the Exosome Isolation Reagent (Thermo Fisher, Waltham, MA, USA) following the manufacturer’s instructions.

Immunoaffinity isolation of plasma small EVs. The Dynabeads Protein G (Invitrogen 10004D) was used for immunoaffinity isolation of plasma small EVs. Antibody cocktails, consisting of antibodies against human CD326 (EpCAM, BioLegend), CD104, c-Met, CD44v6 (Thermo Fisher Scientific), and TSPAN8 (Miltenyi Biotec), were conjugated to magnetic beads with 10 µg of each antibody, total 50 µg antibody diluted in 200 µL PBS, and 100 µL Dynabeads in PBS, rotating for 10 min at room temperature. The conjugated beads were washed once with 200 µL PBS containing 0.2% Tween 20. Small EVs were first isolated from plasma (250 µL per sample) using the Exosome Isolation Reagent (Thermo Fisher, Waltham, MA, USA) following the manufacturer’s instructions. The pooled small EV pellets from each group were dissolved in 500 µL PBS and mixed with the magnetic bead–antibody cocktail, and incubated at room temperature for 30 min with gentle rotation. The resulting complex was washed three times with PBS containing 0.2% Tween 20. Total RNA was extracted from the immunoprecipitated complex and qRT-PCR was performed as described below.

Small RNA library preparation and next-generation sequencing. Total RNA was extracted from small EV pellets using the TRIzol reagent (Invitrogen/Life Technologies, Carlsbad, CA, USA) following the manufacturer’s protocol [12]. RNA concentration was quantitated using a NanoDrop ND-100 Spectrophotometer (NanoDrop Technologies, Wilmington, DE, USA). Twelve plasma small EV samples from patients and matched healthy controls were pooled into four samples, respectively. The RNA samples were sent to Novogene Corporation Inc. for small RNA sequencing using Illumina sequencers and a single-end 50-bp sequencing strategy. More than 10 × 10^6^ reads were collected from each sample, and data were analyzed using a standard protocol at Novogene Corporation Inc. Raw data for each sample were aligned using STAR (Spliced Transcripts Alignment to a Reference) 2.5, and analyzed with DESeq2. Pairwise comparison of the alignment results was performed to identify miRNAs that are differentially expressed. The Benjamini and Hochberg (BH) method was used to adjust multiple testing at significance level of 0.05.

Quantitative real-time reverse transcription polymerase chain reaction. cDNA was synthesized from 70–80 ng of total RNA using the qScript miRNA cDNA Synthesis Kit (Quanta BioSciences, Inc., Beverly, MA, USA) as previously described [20]. An aliquot of cDNA was mixed with Perfecta SYBR Green SuperMix, Quanta Universal PCR Reverse Primer (Quanta, lot#015003), and a sequence-specific forward primer (Table 3) in 20 μL PCR reactions. A synthetic *Caenorhabditis elegans* miR-54 (cel-miR-54) RNA oligonucleotide (Integrated DNA Technologies, Coralville, IA, USA) was spiked into RNA samples as an internal control [28]. PCR reactions were carried on a Bio-Rad CFX 96 Real-Time PCR (Bio-Rad, Hercules, CA, USA) instrument with the following conditions: 95 °C for 2 min, 40 cycles of 95 °C for 5 s, 60 °C for 15 s, and 70 °C for 15 s. Gene expression was calculated using the ΔΔ*C_T_* method: Δ*C_T_* = *C_T_* (target RNA) − *C_T_* (PerfeCTa Human Positive Control for cell or cel-miR-54 for exosome); ΔΔ*C_T_* = Δ*C_T_* (experimental group) − Δ*C_T_* (control group); the fold change = 2^−ΔΔ*CT*^.

Statistics. Statistical analyses were performed using GraphPad Prism 9.5.1 software (La Jolla, CA, USA). One-way ANOVA or a paired Student’s *t*-test was used to determine *p*-values among experimental groups or between matched groups, respectively. A *p*-value of <0.05 was considered statistically significant.

## Figures and Tables

**Table 1:** Clinical features of PDAC patients (PL1–PL12 included for small RNA-seq; PL1–PL20 for qRT-PCR).

Plasma Sample	Age (years)	Sex ^a^	Race ^b^	Diagnosis	Tumor Size Largest Dimension	Stage ^c^
PL1	72	F	W	PDAC	2.5 cm	IIA
PL2	43	F	W	PDAC	1.8 cm	I
PL3	50	F	W	PDAC	1.8 cm	I
PL4	52	M	W	PDAC	1.8 cm	I
PL5	55	M	W	PDAC	1.6 cm	I
PL6	62	M	W	PDAC	2.1 cm	IIA
PL7	60	M	W	PDAC	NA	IIA
PL8	47	F	W	PDAC	NA	IIA
PL9	55	F	W	PDAC	NA	IIA
PL10	67	F	W	PDAC	0.8 cm	I
PL11	50	M	W	PDAC	NA	I
PL12	59	M	W	PDAC	1.8 cm	I
PL13	55	M	B	PDAC	1.8 cm	I
PL14	67	M	W	PDAC	NA	IIA
PL15	63	F	W	PDAC	3.2 cm	IIA
PL16	69	M	W	PDAC	3.1 cm	IIA
PL17	60	F	W	PDAC	2.8 cm	IIA
PL18	67	F	W	PDAC	3.3 cm	IIA
PL19	66	M	W	PDAC	2.6 cm	IIA
PL20	63	M	B	PDAC	1.5 cm	IIA
Mean Age	59.1					

^a^ F, female; M, male. ^b^ W, white; B, black. ^c^ Pathologic TNM tumor staging (American Joint Committee on Cancer Care 7th Edition).

**Table 2:** miRNAs differentially expressed in small EVs of PDAC versus healthy plasma. Small EVs were isolated from plasma of stage I–IIA PDAC patients and matched healthy subjects (n = 12, mean age 56 ± 5 years for PDAC and 54 ± 6 years for healthy subjects, equally divided by gender, all Caucasian). RNAs were isolated and pooled into 4 individual groups for PDAC and healthy controls, respectively. Small RNA sequencing was performed using the Illumina platform SE50. The sequencing data were aligned using STAR (Spliced Transcripts Alignment to a Reference) 2.5, and analyzed with DESeq2. The fold change was converted from log2 fold values (k) using the y = 2k formula.

microRNA	PDAC Mean Reads	Healthy Mean Reads	Fold Change	Adjusted *p*-Value
miR-502	144 ± 116	0 ± 0	371	0.023
miR-18a	186 ± 110	1 ± 1	97	0.011
miR-106a	153 ± 66	1 ± 1	147	0.047
miR-660	445 ± 50	144 ± 3	33	0.011
miR-21	207.461 ± 52,898	125.842 ± 3966	1	0.733

**Table 3:** Primers used for real-time PCR.

microRNA	Primer
miR-502	5′-CATCCTTGCTATCTGGGTGCTA-3′
miR-18a	5′-GCTAAGGTGCATCTAGTGCAGATAG-3′
miR-106a	5′-CAAAAGTGCTTACAGTGCAGGTAG-3′
miR-1246	5′-GCGCGATGGATTTTTGGAGCAG-3′

**Table 4:** Relative miRNA expression in immunoprecipitants of plasma small EVs from PDAC patients versus those from age- and gender-matched healthy controls and colon cancer patients. Small EVs were initially isolated from the plasma of patients with early-stage PDAC and matched colon cancer and healthy subjects. The isolated small EVs (100 µg each) were immunoprecipitated using the magnetic-bead-conjugated antibody cocktail (antibodies against human EpCAM, CD104, c-Met, CD44v6, and TSPAN8). Total RNA was isolated from the immunoprecipitants and qRT-PCR performed for miRNA expression analysis. * *p* < 0.05, ** *p* < 0.01, one-way ANOVA (n = 10–12).

microRNA	Healthy Control Mean ± SEM	PDAC Mean ± SEM	Colon Cancer Mean ± SEM
miR-18a	1.0 ± 0.09	3.05 ± 0.81 *	0.44 ± 0.06
miR-106a	1.0 ± 0.12	4.60 ± 0.79 **	0.74 ± 0.05
miR-196-a	1.0 ± 0.25	1.59 ± 0.45	1.27 ± 0.14

**Table 5:** Colon cancer patients included in this study.

Plasma Sample	Age (years)	Sex ^a^	Race ^b^	Diagnosis ^c^	Stage ^d^
CoP1	65	F	W	CRC	IIA
CoP2	68	F	W	CC	II
CoP3	67	F	W	CC	IIIA
CoP4	41	F	W	CC	IIIC
CoP5	53	F	W	RC	I
CoP6	56	F	W	CC	I
CoP7	43	M	W	CRC	IIA
CoP8	67	M	W	CRC	IIA
CoP9	49	M	W	RC	NA
CoP10	41	M	W	CC	IIIB
Mean Age	55				

^a^ F, female; M, male. ^b^ W, white. ^c^ CRC, colorectal cancer; CC, colon cancer; RC, rectal cancer. ^d^ Pathologic TNM tumor staging (American Joint Committee on Cancer Care 7th Edition).

## Data Availability

Data sharing is not applicable to this article.
